# Powering UAV with Deep Q-Network for Air Quality Tracking

**DOI:** 10.3390/s22166118

**Published:** 2022-08-16

**Authors:** Alaelddin F. Y. Mohammed, Salman Md Sultan, Seokheon Cho, Jae-Young Pyun

**Affiliations:** 1 School of Computing, Gachon University, Seongnam 13120, Korea; 2 European IT Solutions Institute, Dhaka 1216, Bangladesh; 3 Qualcomm Institute, University of California, San Diego (UCSD), 9500 Gilman Drive, San Diego, CA 92093-0436, USA; 4 Department of Information and Communication Engineering, Chosun University, Gwangju 61452, Korea

**Keywords:** UAV, Deep Q-network, unhealthy polluted area, Air Quality Index, IoT

## Abstract

Tracking the source of air pollution plumes and monitoring the air quality during emergency events in real-time is crucial to support decision-makers in making an appropriate evacuation plan. Internet of Things (IoT) based air quality tracking and monitoring platforms have used stationary sensors around the environment. However, fixed IoT sensors may not be enough to monitor the air quality in a vast area during emergency situations. Therefore, many applications consider utilizing Unmanned Aerial Vehicles (UAVs) to monitor the air pollution plumes environment. However, finding an unhealthy location in a vast area requires a long navigation time. For time efficiency, we employ deep reinforcement learning (Deep RL) to assist UAVs to find air pollution plumes in an equal-sized grid space. The proposed Deep Q-network (DQN) based UAV Pollution Tracking (DUPT) is utilized to guide the multi-navigation direction of the UAV to find the pollution plumes’ location in a vast area within a short duration of time. Indeed, we deployed a long short-term memory (LSTM) combined with Q-network to suggest a particular navigation pattern producing minimal time consumption. The proposed DUPT is evaluated and validated using an air pollution environment generated by a well-known Gaussian distribution and kriging interpolation. The evaluation and comparison results are carefully presented and analyzed. The experiment results show that our proposed DUPT solution can rapidly identify the unhealthy polluted area and spends around 28% of the total time of the existing solution.

## 1. Introduction

In recent years, the world witnessed many emergency situations regarding air pollution. These situations are caused by either accidents in industries, natural disasters, or terrorist attacks (e.g., gas leakage in Visakhapatnam, India in May 2020 [1], Fukushima nuclear disaster, Japan, in March 2011 [2]) which can cause a harmful environment for humans and requires a rapid response from decision-makers for evacuation.

A distributed air monitoring network was developed to keep an eye on the density within an area. Internet of Things (IoT) sensors played a vital role as a promising technology for application services monitoring and detecting air quality. However, an enormous number of IoT sensors should be deployed to cover vast areas. These IoT sensors are usually located at a fixed location, sensing locative and temporal variability of air quality [3]. Nevertheless, the existing distributed air monitoring system could be insufficient in large areas to collect air quality data [3]. In response, a new technology covering the large area and improving air quality monitoring is required.

The Air Quality Index (AQI) factor represents how much the air is polluted. The AQI is a global uniform index (scale from 0 to 500) for monitoring the air quality in an area. The index is divided into six ranges, where the range from 151 to 200 is denoted as “Unhealthy” in which general people, as well as sensitive people, could be affected badly [4]. It has to be noted that [4] reports that the AQI barely exceeds the 200 level in the United States. In any AQI monitoring application, the environment is a significant property, where the value of AQI can be the worst suddenly. A sub-optimized solution is needed for monitoring the AQI environment effectively.

In research and academia, deep learning has been widely used in different areas since the evaluation of hardware equipment. Some existing solutions utilize artificial neural networks (ANNs) to predict air pollution. For instance, the authors in [5] claimed that combining numerical models and real-time data in data assimilation techniques presented an outstanding possibility to produce a precise air pollution map. However, because of the air pollution plume dynamics, a circumstantial locative provisional settlement in an emergency event is highly required to act effectively in real-time.

Deep reinforcement learning (Deep RL) is a promising machine learning-based method, which is advanced from the traditional RL method (Q-table) by approximating the sub-optimal action with a deep neural network [6]. The main advantage of Deep RL is that it can decide the best action after interacting with an unknown air-polluted environment instead of depending on many historic resources.

An Unmanned Aerial Vehicle (UAV) is a small aircraft (drone) that can be controlled remotely or pre-programmed. Many applications use UAVs for military, surveillance, search and rescue, localization, remote sensing, and telecommunications. Moreover, the UAV can be used for air pollution monitoring and tracking applications. For example, the authors in [7,8,9,10,11] presented air monitoring systems using UAVs to measure air quality and pollution concentration in a predefined area utilizing different types of sensors. Another example in [12,13] developed a pollution source tracking algorithm for multi-UAVs, including strategies to prevent collisions between the UAVs.

In general, UAVs monitor and track the air-polluted environment by navigating and sensing from one area to another. To control the UAV navigation effectively, several navigation methods have been introduced (e.g., spiral [14,15], and billiard [3,7,12]). In the spiral navigation pattern, the movement focuses on a central spot with a chain of circular trajectories revolving around the center. On the other hand, in the billiard navigation pattern, the navigation starts from a corner of the selected area and then covers the entire region by moving back and forth. The authors in [15,16] claimed that the spiral UAV navigation pattern takes a significantly shorter time compared to the billiard navigation pattern to cover the entire area. However, those existing solutions require a long time to track the source of air pollution.

Therefore, It is essential to utilize the UAV resources efficiently for a short time to track single or multiple polluted areas. To overcome the issue of limited navigation time in a complex polluted environment, we propose a Deep Q-network (DQN) solution. The following are the detailed contributions to this paper:

1. We introduce the DQN solution to track unhealthy polluted environments. We mainly focus on finding the unhealthy polluted area (i.e., AQI > 150) from the air-polluted environment using multi-navigation patterns.
2. We apply a long short-term memory (LSTM) combined with DQN to predict sub-optimal UAV navigation patterns with the measurement time-sequential AQI data.
3. The proposed solution selects multi-pattern navigation (i.e., forward, down-right, down-left, zigzag) in the action space of the DQN.
4. The UAV agent can decide a specific navigation pattern chosen by the LSTM Q-network at the particular situation or state of the environment to reduce the UAV navigation distances and time.

We present the system model and evaluate the results of our proposed solution by comparing it with the spiral solution in both single and multiple polluted environments. Our simulation results show an outstanding performance in finding the unhealthy area with minimal navigation time and distances compared to the current solution.

The remainder of this paper is organized as follows. A description of the related work is provided in Section 2. Section 3 presents the system preliminaries. Section 4 depicts the proposed DQN-based UAV navigation system’s simulation setup, then shows the simulation results, detailed analysis, and comparison. Finally, Section 5 presents the conclusions and future directions of the research work.

## 2. Related Work

From recent research, we can classify the research effort regarding utilizing the UAVs for air pollution monitoring into two categories: (1) monitoring an area, and (2) finding the polluted area. The following subsections summarize these research efforts.

### 2.1. Monitoring AQI the Entire Area

Nowadays, various UAV-related applications and services have been introduced for air pollution monitoring, for instance [7,8,9,10,11]. The authors in [7] employed UAVs using lightweight air pollution sensors for measuring particle matter and ultrafine particles. The experimental results showed good measurement accuracy regarding horizontal and vertical variations in ultra-fine matter concentrations. The authors in [8] proposed a vision-based UAV technique to monitor the AQI. An onboard high-definition camera was used to capture the aerial panoramic image along with various directions, and the UAV collected the AQI from all directions (360-degree images). Under different air conditions, the targeted area was divided into disjointed hexagonal grids to collect the AQI data effectively. Subsequently, authors in [8] proposed a feature-based image matching method to recognize the AQI from the images (using Haze Model and Medium transmission). The authors claimed that their results presented a good AQI observation accuracy with low power consumption.

The authors in [9] utilized a Quadrotor UAV to monitor air quality based on IoT technology. The UAV was integrated with sensors used to detect various gases and temperatures. The position of the monitored area was recognized using the Global Positioning System (GPS), and the measured data was transferred into two servers, a web server and a mobile SMS server. Authors in [10] showed a new air quality measurement to prevent atmospheric ground-based volatile organic compound pollution. The authors designed a mission planning strategy to obtain the trajectory of the UAV during data collection. Fine characterization used in [10] system effectively reduces measurement errors.

### 2.2. Tracking Unhealthy Polluted Area

Tracking the source of air pollution is a demanding application [12,13,17]. For instance, gas leakage may cause massive destruction when a lack of proper gas observation is not performed. Thus, finding the source of gas leakage is essential to prevent harmful circumstances.

Authors in [12] utilized multi-UAVs for tracking a source of the gas leakage by combining the particle swarm optimization algorithm and artificial potential field algorithm.

The authors used an ad hoc network to avoid collisions between UAVs for high-quality communication. However, the multi-UAV in [12] system could not support a complex multi-pollution environment. Moreover, authors in [13] proposed multi-UAV source tracking of air pollution by utilizing particle swarm optimization. The objectives in [13] were to avoid a multi-UAV collision while finding the source of air pollution.

Finding the source of air pollution quickly is beneficial and significant. However, the existing solutions use more resources (e.g., multi-UAV) and consume time to find the unhealthy area. This paper focused on utilizing DQN on the UAV to reach the unhealthy polluted area within a short time rapidly. Table 1 represents a comparison of different existing methods along with advantages and disadvantages.

## 3. DQN-Based UAV Pollution Tracking (DUPT) Methodology

The proposed DQN-based UAV Pollution Tracking (DUPT) approach is designed to quickly track and find unhealthy polluted locations in a big area. In this paper, the unhealthy area is the area with an AQI value greater than 150 (as classified in [4]). The following subsections describe the methodology of our proposed DUPT.

### 3.1. System Overview

**Figure 1:**
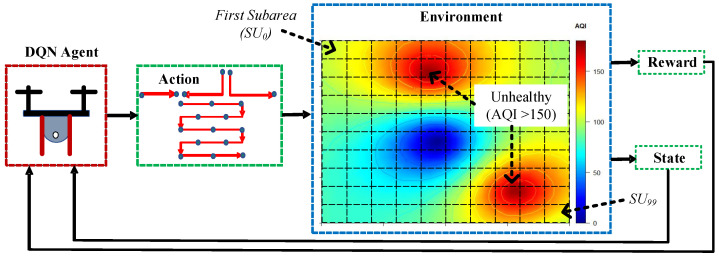
DQN UAV agent for tracking unhealthy areas (AQI > 150).

Figure 1 illustrates the AQI environment where one UAV is allocated to find one or more unhealthy polluted areas. We suppose that the environment is divided into multiple subareas ($SU_{0},SU_{1},\ldots ,SU_{M-1}$), and the distance between two subareas is *S* [*m*] (similar to [15,16]). Thus, the environment contains *M* subareas. The UAV navigates, senses, and calculates the AQI value at the subarea. Moreover, we assume that the position of the AQI observation is in the middle of a subarea. The size of the environment is defined as X×Y m. We can estimate the *M* by assuming *X* is equal to *Y* in Equation (1).

(1) $$\;M={\left(\frac{X}{S}\right)}^{2}.$$

The deployed UAV is assumed to fly with a certain speed from $SU_{m}$ to $SU_{n}$ (*m* and *n* are the subarea number). A UAV usually cannot always move at the same speed because of wind and other obstructions [20,21]. As a result, we used an average flying speed, $av_{speed}$ m/s for the performance evaluation. The UAV travels for a specific distance $d_{i}$ at the step *i* (e.g., 200 m, 500 m, 1000 m), leading the movement from $SU_{m}$ to $SU_{m+n}$, where *n* is the total number of step distance. When the UAV reaches a subarea, it has to hover, sense, and sample the AQI value. The main objective is to find out the unhealthy area (AQI value is greater than 150) in a short time. For evaluation, we estimate the following as shown in Equations (2) and (3),

(2) $$\;T_{Nav}=\sum_{M}T_{col}+T_{tra},$$

(3) $$\;T_{tra}=\left(\sum \frac{d_{i}}{av_{speed}}\right),$$

where $T_{Nav}$ is the total navigation time including the time for traveling ($T_{tra}$) the *n* subareas and time of collecting the AQI values ($T_{col}$) for each subarea.

As shown in Figure 1, the DUPT takes state ($s_{t}$) as an input and decides the appropriate action ($a_{t}$) at a particular time (*t*). After taking action, the DUPT agent interacts with the environment, achieves reward feedback ($r_{t}$), and jumps to the next state ($s_{t+1}$) for performing a new action. The following subsection describes this process of DUPT.

### 3.2. State Space

We assume that the unhealthy polluted area is unknown and can be anywhere in a particular environment. For the UAV navigation decision, the UAV agent needs AQI measurement of the current subarea (e.g., $SU_{C}$) and the surrounding subareas information (e.g., $SU_{A}$ and $SU_{B}$).

**Figure 2:**
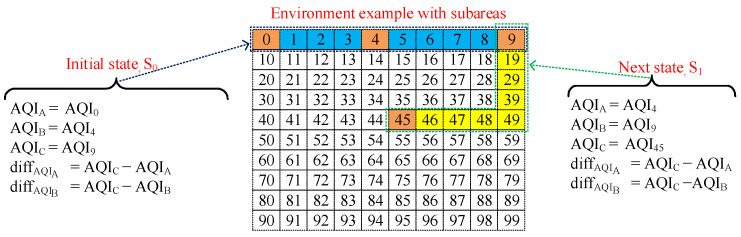
Test environment and measured factors for DQN UAV navigation.

Moreover, the proposed DUPT calculates the difference between the previous two AQIs and the current AQI values. Figure 2 is an example of an environment with 100 subareas indexed from $SU_{0}$ to $SU_{99}$. The DUPT initially starts from $SU_{0}$ and traverses the surrounding location (i.e., $SU_{4}$ and $SU_{9}$). The state space in DUPT has factors such as $s_{t}$ = {$SU_{A},SU_{B},SU_{C},AQI_{A},AQI_{B},AQI_{C},diff_{AQI_{A}},diff_{AQI_{B}}$}. The description of these factors in the state space is given in Table 2.

### 3.3. Action Space

Designing a better action space is essential in the DQN-based Deep RL method for sub-optimal performance. The proposed DUPT aims to search the unhealthy polluted area as shown in Figure 1. However, the UAV agent does not monitor all subareas in the vast environment due to limited battery life. As a result, the proposed DUPT agent skips some subareas to reduce the overall flying distance and time. Following that, DUPT combines multiple navigation patterns for the action space, $A_{P_{K}}$ = {$A_{P_{1}},A_{P_{2}},A_{P_{3}},A_{P_{4}}$} in a such way that the DUPT can skip some SUs which have similar AQIs with the neighboring subareas. Therefore, the DUPT can reduce the overall UAV navigation time and distance to reach unhealthy areas. We used *K* as a number of navigation patterns, where *K* = 4 in our work.

**Figure 3:**
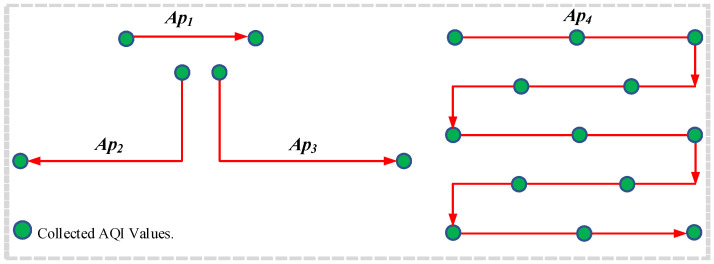
DUPT Navigation action patterns ($A_{P1},A_{P2},A_{P3},A_{P4}$) of proposed DUPT agent.

Figure 3 represents different navigation patterns ($A_{P_{K}}$) that proposed DUPT can choose based on a state condition. Note that the distance from the current AQI collection position to the next position varies on the chosen navigation patterns.

In DUPT, we compare three different AQIs ($AQI_{A}$, $AQI_{B}$, $AQI_{C}$) at each subarea to determine a navigation pattern to be applied to the current state space (AQI observation environment).

For example, if the current AQI ($AQI_{C}$) is bigger than any of the old AQIs ($AQI_{A}$ or $AQI_{B}$) as well as the differences, $diff_{AQI_{A}}$ and $diff_{AQI_{B}}$ are less than its threshold (thres), then the UAV navigation pattern is chosen as $A_{P_{1}}$. On the other hand, if $AQI_{C}$ is bigger than any of the $AQI_{A}$ or $AQI_{B}$ but both differences are larger than the thres, then there will be a possibility that an unhealthy area exists. Therefore, the DUPT UAV agent needs to traverse surrounding areas by the zigzag pattern action ($A_{P4}$) to collect more AQI values to find the unhealthy area. The conditions for choosing the UAV navigation patterns are described as follows:

$$act_{scene}=\left\{\begin{array}{cc} 1\;\;\;\;\;\mathrm{if} & \left\{\begin{array}{c} \{(AQI_{C}>AQI_{A})||(AQI_{C}>AQI_{A})\}\;\&\;\\ \left\{(diff_{AQI_{A}}<thres)\;\&\;(diff_{AQI_{B}}<thres)\right\} \end{array}\right.\\ 2\;\;\;\;\;\mathrm{if} & \left\{\begin{array}{c} \{(AQI_{A}\leq AQI_{B})\;\&\;(AQI_{C}\leq AQI_{A})\;\&\;(AQI_{C}\;\&\;AQI_{B})\}||\\ \left\{(AQI_{B}<AQI_{A})\;\&\;(AQI_{C}<AQI_{A})\;\&\;(AQI_{C}\leq AQI_{B})\right\} \end{array}\right.\\ 3\;\;\;\;\;\mathrm{if} & \left\{\{(AQI_{C}=AQI_{A}=AQI_{B})\right\}\\ 4\;\;\;\;\;\mathrm{if} & \left\{\begin{array}{c} \left\{\begin{array}{c} \{(AQI_{C}>AQI_{A})||(AQI_{C}>AQI_{B})\}\;\&\;\\ \left\{(diff_{AQI_{A}}\geq thres)\;\&\;(diff_{AQI_{B}}\geq thres)\right\} \end{array}\right.\\ ||\{(AQI_{C}>150)\} \end{array}\right. \end{array}\right.$$

The values 1, 2, 3, and 4 of the above formula ($act_{scene}$) represent the four UAV navigation patterns $A_{P1}$, $A_{P2}$, $A_{P3}$, $A_{P4}$, respectively, as already shown in Figure 3. Note that the DUPT UAV system selects an action ($a_{t}$) in an epsilon-greedy manner. During the epsilon period, the DUPT agent randomly selects one of the *K* actions to explore the environment. Following that, the agent starts exploiting the environment after completing the epsilon period. During the exploitation, the UAV agents decide on a sub-optimal action by using a greedy strategy from our proposed DQN (*Y*) as shown in Equation (4).

(4) $$greedy_{a_{t}}=\arg\max\left(Q\left(s_{t},a_{t};Y\right)\right).$$

### 3.4. Reward Space

In the proposed DUPT, we used a binary reward strategy [22] to evaluate the performance of the DQN-based UAV agent. The main reason for using binary reward is that it is simple to estimate without any computational complexity. The proposed DUPT receives the reward ($r_{t}$) as feedback at time *t* according to Equation (5).

(5) $$r_{t}=\left\{\begin{array}{cc} 1 & \mathrm{if}\;A_{P_{k}}\;=\;act_{scene}\\ 0 & \mathrm{otherwise}, \end{array}\right.$$

where $A_{P_{k}}$ is the navigation pattern as shown in Figure 3.

### 3.5. Training Methodology

In any DQN-based system, training is the most crucial task to achieve better performance. As a result, to obtain a better and sub-optimal action at the DUPT, we utilized two different strategies (similar to [6]): (a) experience replay and mini-batch (ERM), and (b) separate target Q-network (STQN). The following subsections describe ERM and STQN utilization in the DUPT.

#### 3.5.1. Experience Replay Memory and Mini-Batch (ERM)

In DUPT, the UAV agent always learns by trial and error through an interactive process with the environment. Therefore, the system needs to utilize the previous experiences more than one time. To reuse the same experience, ERM is employed for storing each time (*t*) experience. The experience includes all the essential transitions of the UAV agent. Each transition consists of the current state ($s_{t}$), current action ($a_{t}$), current reward ($r_{t}$), and the next state ($s_{t+1}$).

However, as the training period increases, the number of experiences also increases sequentially. As a result, the size of the memory could be more prominent. If we train all experiences together without any randomness, the system can form instability during the training session. To overcome this problem, we need to reduce the correlation between the experiences by training a specific size of experiences instead of training all experiences together.

**Figure 4:**
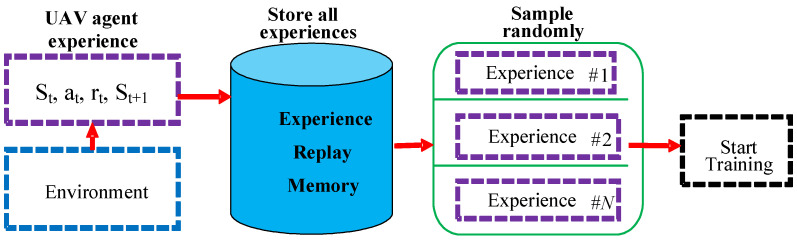
ERM architecture in DUPT.

The mini-batch strategy can extract a set of experiences randomly to increase the decorrelation between the experiences; thus, the DUPT can provide a better sub-optimal action. Figure 4 represents the architecture of DUPT-ERM for better understanding.

#### 3.5.2. Separate Target Q-Network (STQN)

STQN is another approach for improving the performance of the DQN system. A key objective in DQN is to minimize the estimation loss as much as possible. The estimation loss was obtained by comparing both predicted Q-values and target Q-values. In the DUPT system, two different Q-networks (θ and $\theta^{\prime }$) were utilized for predicting Q-values and estimating target Q-values, respectively. STQN assists to acquire optimized loss value observed by Equation (6), where *Y*, $Y^{\prime }$, and γ denote the predicted Q-values from θ Q-network, target Q-values from $\theta^{\prime }$ target Q-network, and discount factor ∈[0,1], respectively.

(6) $$\;L\left(\theta \right)={\left(Y^{\prime }-Y\right)}^{2}.$$

(7) $$\;Y=Q(s_{t},a_{t};\theta ).$$

(8) $$\;Y^{\prime }=r_{t}+\gamma \max\left(Q\left(s_{t+1},a_{t+1};\theta^{\prime }\right)\right).$$

### 3.6. Q-Network in DUPT

**Figure 5:**
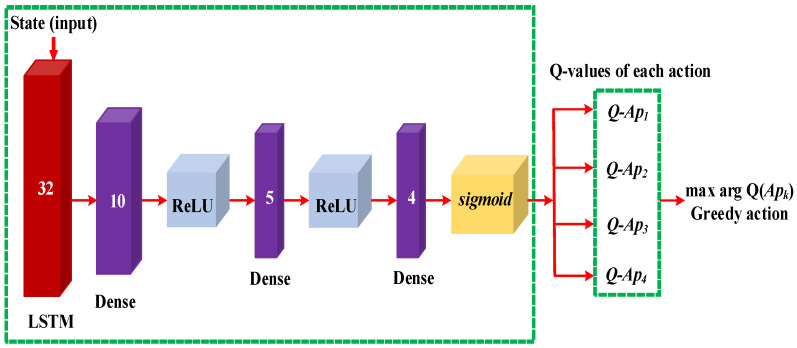
Q-network in DUPT.

In our proposed DUPT, we employ a combination of LSTM and Dense for Q-network predicting action Q-values as shown in Figure 5. The LSTM is a part of the Recurrent Neural Network (RNN) utilized for time-series forecasting. The LSTM consists of the different gate operations (i.e., forget, input, cell, output). By utilizing these gates, the LSTM can prioritize which information should be stored or removed. In our designed state space, the values are sequentially updated, which means that it is time-dependent. Consequently, the LSTM Q-network works effectively in terms of sub-optimal navigation pattern selection.

Dense is a Fully Connected Neural Network (FCNN) that is also employed for time-series-based state space. We integrate LSTM and Dense together to obtain the stable performance of the DUPT. Figure 5 shows the architecture of the proposed Q-network, where the state is the input layer. In this Q-network, LSTM is employed as the first hidden layer (with the size of 32) while the other three layers (with the size of 10, 5, and 4) are Dense based FCNN. Note that the first two Dense layers are assumed as hidden layers and the last Dense layer is the output layer. Moreover, the sizes 10 and 5 are selected in a trial and error manner for the first and second Dense layer, respectively. The size of the final dense layer is 4 because we have four actions in the action space as already presented in Figure 3. For the activation function, we use “Relu” in each layer, except the first and last layers to neglect the negative weighted values. Finally, we utilize the “sigmoid” activation function to bound the Q-values between 0 and 1. The operation of our proposed system is represented by Algorithm 1.
   **Algorithm 1:** DUPT Algorithm      
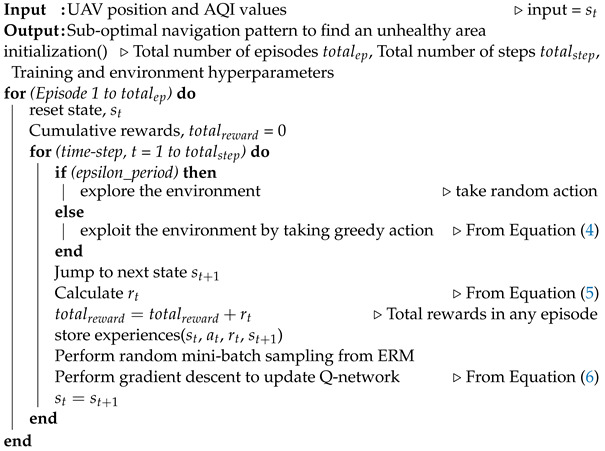


## 4. Simulation and Result Analysis

### 4.1. DUPT Simulation Setup

#### 4.1.1. Environment and UAV Setup

To evaluate the performance of our proposed DUPT, it is crucial to generate polluted environments with AQI distribution. The size of each environment is 4000 m × 4000 m with a subarea size of 100 m × 100 m, similar to [15,16]. A total number of 1600 subareas ($SU_{0}$∼$SU_{1599}$ can exist based on Equation (1)) at each environment. Further, The AQI values are distributed between 0 and 180, and the environments are designed according to a well-known Gaussian distribution and kriging interpolation similar to [15,16]. Note that, The area showing AQI greater than 150 is considered an unhealthy polluted area.

**Figure 6:**
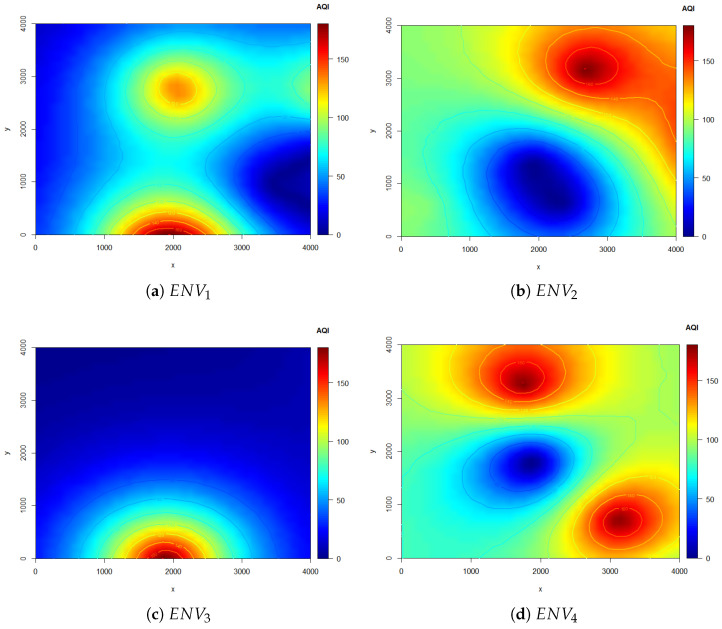
Testing Environments for the proposed DUPT.

Figure 6 represents four 2D (X-Y plane) different environments including one unhealthy polluted area (Figure 6a–c), and two unhealthy polluted areas (Figure 6d). Details of the environments and UAV parameters are given in Table 3.

#### 4.1.2. DUPT Training Hyperparameters

To design a sub-optimal Q-network, we initialized some hyperparameters (e.g., optimizer, learning rate, loss, and so forth). These hyperparameters control the characteristics of the Q-network to provide the best action. Table 4 describes the training hyperparameters of the proposed DQN in detail.

Adam [23] is one of the most popular and effective optimizers for LSTM-based RNN because the Adam optimizer can tune the weights of the LSTM very efficiently during the training period. Moreover, we used the categorical crossentropy loss function to estimate the error rate between predicted Q-values and target Q-values as already described in Equation (6). The main reason for selecting categorical crossentropy is that the size of our action space is more than two [24]. The rest of the hyperparameters are achieved by trial and error strategy during the training period. Note that the discount factor’s value, γ, maximum epsilon, minimum epsilon, and epsilon decay are always assumed inclusively between 0 and 1 [6].

### 4.2. Simulation Result Analysis

In our simulation, we ran the proposed model on TensorFlow 2.2.0 [25] API under python 3.5 [26]. With Keras library [27], we evaluated our proposed DQN performance in terms of two perspectives: (1) navigation mapping of proposed DUPT, and (2) mobility (i.e., UAV coverage, traveling time, AQI sensing, and total time to reach the unhealthy polluted area). Since the environment could include one or more AQIs higher than 150, we configured the proposed DUPT under two different setup conditions.

A. **$DUPT_{terminate}$**: In this setup condition, the UAV agent will stop traveling if the UAV reaches an unhealthy area (i.e., AQI > 150) to reduce the distance coverage and time.
B. **$DUPT_{continue}$**: In this setup condition, the UAV agent continues its traveling until it reaches the maximum time-step threshold (i.e., 200) in an episode even though the system reaches the unhealthy polluted area.

To compare our proposed DUPT with existing solutions, we implemented the UAV movement under the spiral solution described in [15,16] for all the environments. Furthermore, when we compared $DUPT_{terminate}$ against the spiral solution, we terminated the simulation in the spiral navigation pattern after the UAV reaches the unhealthy area. On the other hand, we kept the UAV in the spiral solution to search for the second unhealthy area, when $DUPT_{continue}$ is used for the comparison with the spiral solution.

#### 4.2.1. Single Unhealthy Area ($DUPT_{terminate}$)

In this subsection, we plot the navigation pattern utilized by our DUPT solution under the setup of $DUPT_{terminate}$. Then, we compare the results with the spiral solution.

**Figure 7:**
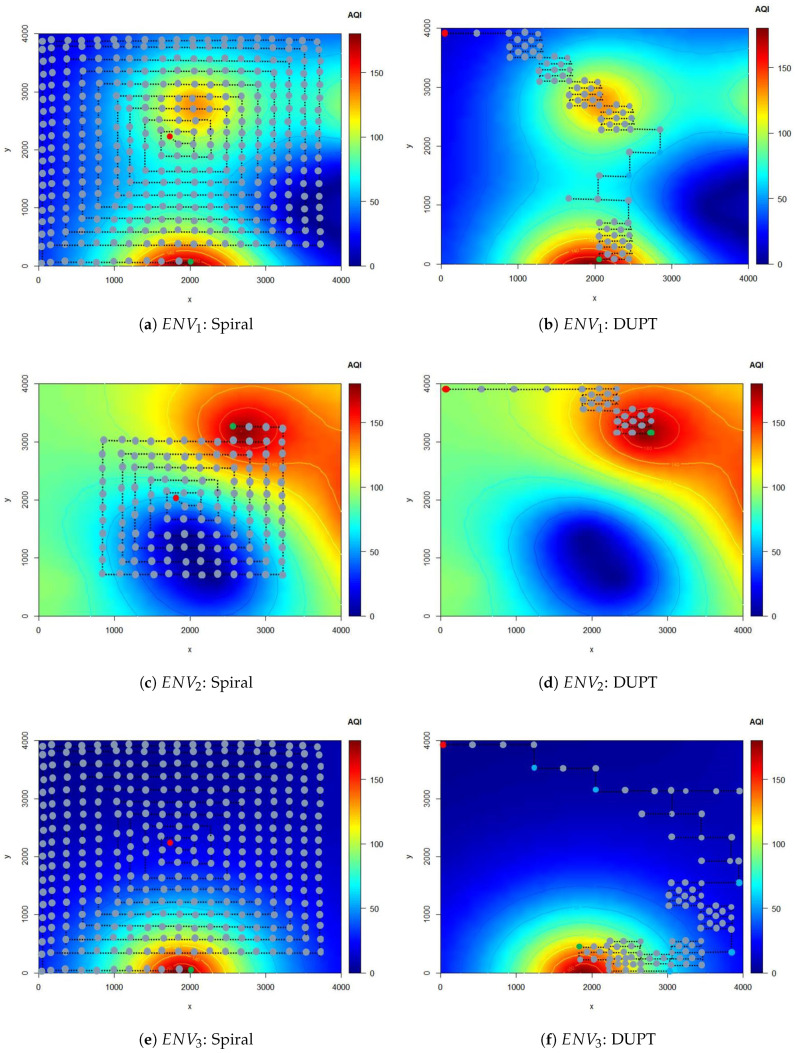
Navigation results to find the unhealthy polluted area.

Figure 7 represents the navigation mapping of both the spiral solution and DUPT for $ENV_{1}$, $ENV_{2}$, and $ENV_{3}$ of Figure 6. It can be observed that our proposed DUPT reaches the unhealthy polluted area successfully for each in the presented environment. We can observe in Figure 7b,d,f that the DUPT starts with $A_{P_{1}}$ step action to collect information from the environments and decides the next action. In Figure 7b, we can see that the DUPT repeats the $A_{P_{4}}$ step action four times consecutively. The reason behind this is that the AQI difference threshold (thres) is exceeded. After this fine searching for the unhealthy area, the step actions $A_{P_{1}}$, $A_{P_{2}}$, and $A_{P_{3}}$ are performed because small AQIs are observed during $A_{P_{4}}$ searching time.

In the end, DUPT performed $A_{P_{4}}$ action and found the area that has AQI > 150 and terminates searching.

In Figure 7d ($ENV_{2}$), the DUPT did not take much time to complete the search because the target was near the start position of the UAV agent. It took the UAV agent to utilize $A_{P_{1}}$ step action twice at the beginning, and $A_{P_{4}}$ step action twice at the end. We can conclude that it is crucial to select a proper start position for the UAV to rapidly find the most harmed environment for human life.

In Figure 7f ($ENV_{3}$), the environment is very common in rural areas wherein in most locations, the AQI values are very similar. However, in this environment, there is one location with an unhealthy area, but its location is far from the starting point of the UAV agent. Thus, the UAV agent in Figure 7f spends time navigating using $A_{P_{1}}$, $A_{P_{2}}$, and $A_{P_{3}}$ step actions until it found a difference in the AQI values. The UAV agent uses $A_{P_{4}}$ twice, then $A_{P_{2}}$ and $A_{P_{4}}$ until it reaches the unhealthy location.

On the other hand, in the spiral solution, there is no intelligence in searching the environment. The UAV must travel in a predefined route and search every predefined position to find the unhealthy area searching the unhealthy area value as shown in Figure 7a,c,e. Therefore, the UAV might spend more time searching in all areas. The spiral solution might be good for environment monitoring; however, it might fail in a vast area due to the battery life restrictions of the UAVs.

**Figure 8:**
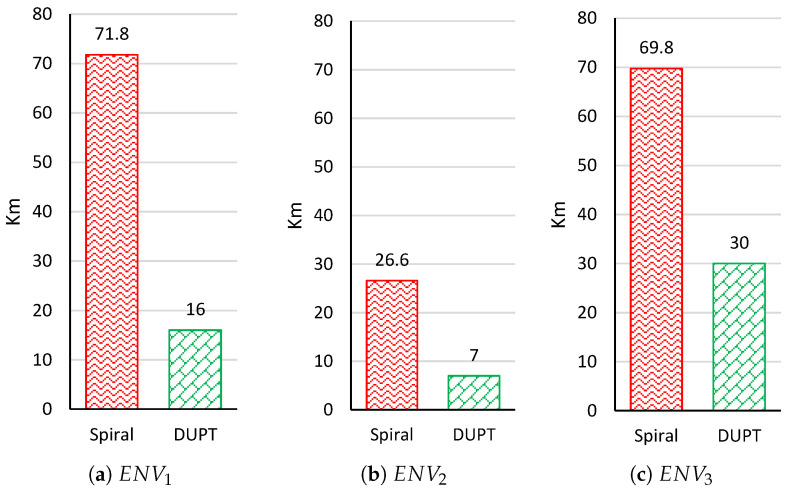
Flying Distance results at $ENV_{1}$, $ENV_{2}$, and $ENV_{3}$.

Figure 8 presents the flying distance comparison for $ENV_{1}$, $ENV_{2}$, and $ENV_{3}$. The result shows that our DUPT solution witnessed different flying distances. The lowest flying distance (7 km) of DUPT is found in $ENV_{2}$, whereas the unhealthy area is found in $ENV_{3}$ (30 km). The difference is caused by how far the starting point of the UAV is from the unhealthy polluted area in the environment. Compared to the spiral solution, we can see that our DUPT flies for a short distance to find the unhealthy area.

In this paper, we define two different time durations the UAV can experience during tracking the unhealthy areas: (1) flying time, measured using Equation (3), which is the total time the UAV can spend flying from one point to another point, and (2) sensing time, which is the total time the UAV can spend to collect the AQI.

**Figure 9:**
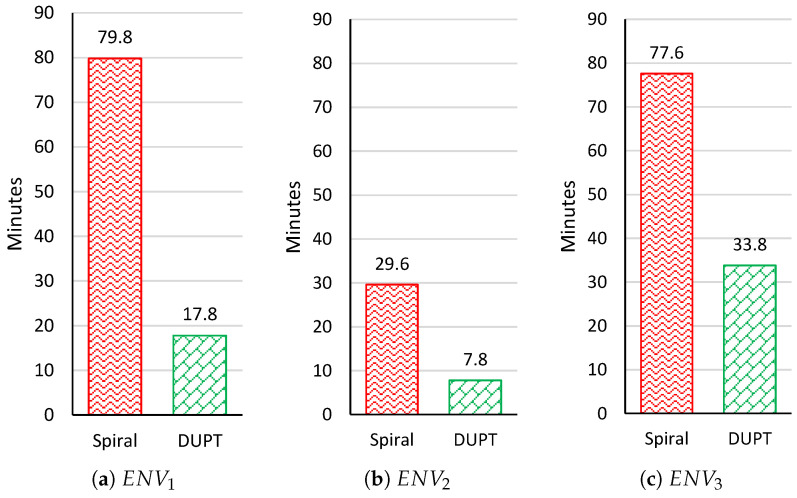
Flying time results at $ENV_{1}$, $ENV_{2}$, and $ENV_{3}$.

Figure 9 depicts the flying time for spiral way and DUPT. In this paper, we assume that the UAV’s average speed ($av_{speed}$) is 15 m/s. Results show that using DUPT; the UAV can spend a very short time than the spiral solution. The results are correlated with the results in Figure 8. We can intentionally conclude here that the UAV can spend the shortest time finding the unhealthy area when the location is nearby the starting point.

**Figure 10:**
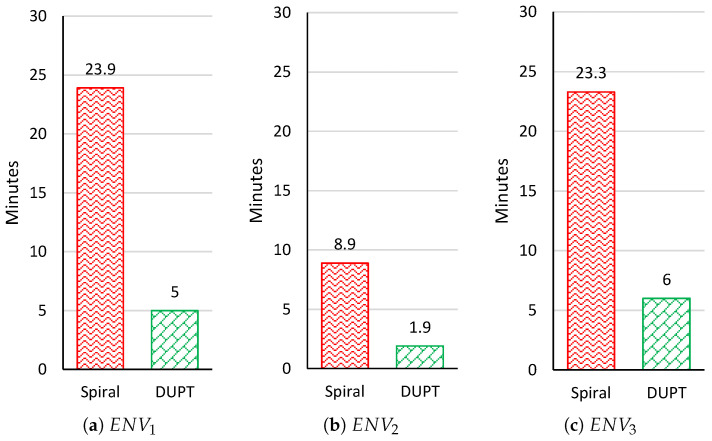
Total sensing time results at $ENV_{1}$, $ENV_{2}$, and $ENV_{3}$.

We consider in this paper that the sensing time for a subarea is 4 s (similar to [15,16]). Figure 10 shows the total sensing time in DUPT did not exceed 6 min. However, the spiral solution shows that it requires a much longer sensing time. Since the UAV during sensing is hovering, it can consume more power than flying. Spending a long time in sensing and calculating the AQI can drain the UAV battery rapidly. The outstanding DUPT solution can help to cover a wide area and can still keep saving the battery of the UAV compared to the spiral solution. It has to be noted that calculating the energy consumption is not considered in this paper.

**Figure 11:**
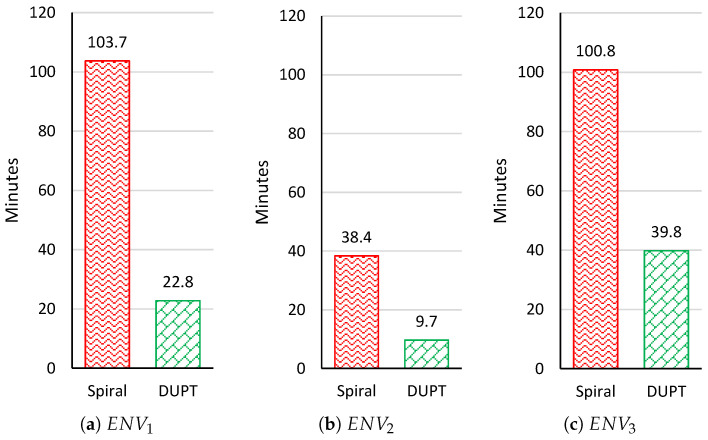
Total time results at $ENV_{1}$, $ENV_{2}$, and $ENV_{3}$.

The total time the UAV spent from the starting point until it reached the goal is presented in Figure 11. We can see that in the spiral solution, the UAV spent more than 100 min until it reached the goal. However, in DUPT, the worst time was 39.8 min in $ENV_{3}$. To explain what happened in $ENV_{3}$, we need to recall Figure 7f. We can observe from Figure 7f that the environment has almost the same AQI value in most of the areas. Thus, the UAV spends more time (using action navigation patter $A_{P_{1}}$, $A_{P_{2}}$, and $A_{P_{3}}$) until it discovered AQI differences and then it uses action navigation pattern $A_{P_{4}}$. This is also reflected in Figure 10c.

Moreover, since the polluted environment is different in every case, we can conclude that the unhealthy location area relative to the starting point of the UAV is very significant for shorting the time of finding the unhealthy area. Furthermore, we can depict that our solution can find the unhealthy area much faster. From Figure 11, we can conclude that the proposed DUPT spends 28% of the total time of the spiral solution. This means that our proposed DUPT is around two times faster than the spiral solution.

#### 4.2.2. Multiple Unhealthy Area Using $DUPT_{continue}$

**Figure 12:**
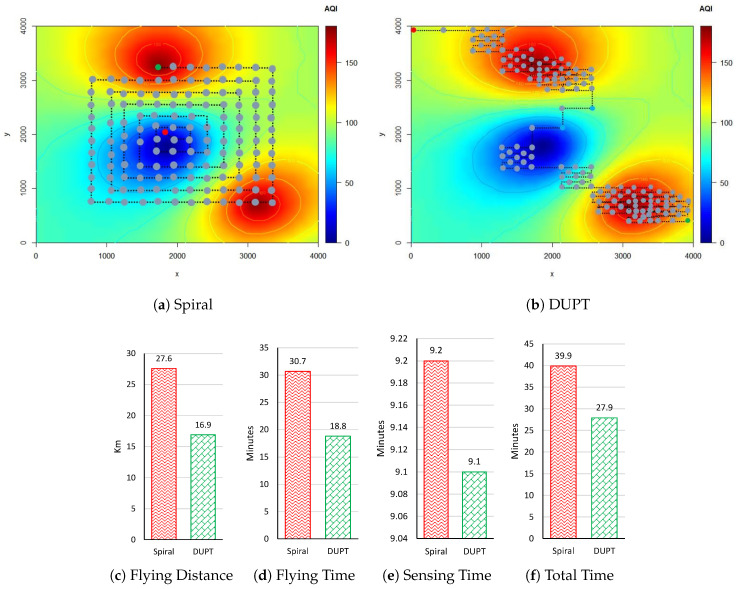
Simulation results at $ENV_{4}$.

In this section, we are interested in evaluating the $DUPT_{continue}$ solution and comparing the results with the spiral solution. Figure 12b depicts that DUPT successfully reaches the expected destination with two unhealthy areas. DUPT chooses different action patterns without stopping its traveling before 200-time steps. As a result, the proposed DUPT collects more AQI values in the AQI-based unhealthy environment.

## 5. Conclusions

In this paper, we have utilized DQN to assist the UAV in finding unhealthy areas by providing a proper navigation action in a polluted environment. The main goal of the proposed DUPT is to find the unhealthy polluted area with sub-optimal time and distance due to the limited battery life span of the UAV. As the unhealthy polluted area can exist anywhere in the environment, the UAV collects more AQI value by moving surrounding areas when it observes a significant difference between the current AQI and two previous consecutive AQIs. If the difference is larger than a threshold, then the UAV traverses with the action designated action to reach the unhealthy area. The proposed scheme has been evaluated in four different environments. The illustrated results show that the UAV can reach the position with the unhealthy area by intelligently traversing a short path. The results have revealed that our proposed solution can achieve the goal two times faster than the spiral solution.

Since our proposed solution is mainly designed for tracking the unhealthy area, it might not be enough for monitoring the entire selected area. However, the proposed DUPT can be satisfied by searching one or two unhealthy polluted areas. In our future work, we will extend our work to be more intelligent in various air-polluted environments and include a monitoring function.

## Figures and Tables

**Table 1:** Comparison of the proposed method with the other methods.

Study	Purpose	Method	Advantages	Disadvantages
[18,19]	Monitoring	Arduino sensor modules	Air quality monitoring system	Not used for tracking. Navigation pattern not defined
[12]	Tracking	Particle swarm optimization and artificial potential field	Used multi-UAV under ad hoc network to avoid collision	Could not support in the complex multi-pollution environment
[15,16]	Tracking and Monitoring	Spiral Pollution-driven UAV Control	Covered the all polluted area	Navigation time is large due to spiral navigation pattern
DUPT	Tracking	DQN	Find out the unhealthy area within a short duration of time in both single and multi-pollution environments	Monitoring the air pollution environment with multi-UAV is out of this research scope

**Table 2:** State space description.

Symbols	Description
$SU_{A}$	previously selected subarea before $SU_{B}$ of UAV
$SU_{B}$	previously selected subarea before current subarea $SU_{C}$ of UAV
$SU_{C}$	current subarea of UAV
$AQI_{A}$	previous AQI value before $AQI_{B}$
$AQI_{B}$	previous AQI value before current $AQI_{C}$
$AQI_{C}$	current AQI value
$diff_{AQI_{A}}$	difference between $AQI_{A}$ and $AQI_{C}$
$diff_{AQI_{B}}$	difference between $AQI_{B}$ and $AQI_{C}$

**Table 3:** Environment and UAV parameters.

Parameters	Value
Size of a environment	4000 m × 4000 m [15,16]
Total number of subareas	1600
Large AQI difference threshold (thres)	10
Number of UAVs in the environment	1
UAV average speed ($av_{speed}$)	15 m/s
UAV step distance for action $A_{P_{1}}$	500 m
UAV step distance for action $A_{P_{2}}$ and $A_{P_{3}}$	1000 m
UAV step distance for action $A_{P_{4}}$	200 m
AQI collection time ($T_{col}$)	4 s [15,16]

**Table 4:** Training hyperparameters.

Hyperparameters	Value
Optimizer	Adam [23]
Loss	Categorical crossentropy [24]
Batch Size	32
Size of experience replay memory (*E*)	1000
Learning rate (*∂*)	0.0001
Discount factor (γ)	0.7
Maximum epsilon	1
Minimum epsilon	0.001
Epsilon decay	0.995
